# Genome Editing among Bioethics and Regulatory Practices

**DOI:** 10.3390/biom12010013

**Published:** 2021-12-22

**Authors:** Mauro Mandrioli

**Affiliations:** Department of Life Sciences, University of Modena and Reggio Emilia, Via Campi 213/D, 41125 Modena, Italy; mauro.mandrioli@unimore.it

**Keywords:** genome editing, bioethics, regulatory practices

## Abstract

In the last decade, genome editing technologies became very effective and several clinical trials have been started in order to use them for treating some genetic diseases. Interestingly, despite more than 50 years of discussion about the frontiers of genetics in human health and evolution, the debate about the bioethics and the regulatory practices of genome editing is still far from satisfactory answers. This delay results from an excessive emphasis on the effectiveness of the genome editing technologies that is relevant for the regulatory practices, but not at a bioethical level. Indeed, other factors (such as accessibility and acceptability) could make these techniques not accepted at the bioethical level, even in the presence of their 100% effectiveness.

## 1. Introduction

In the last two decades the availability of completely sequenced human genomes prompted several new studies and the development of first attempts of gene therapy, based on the transfer of functional copies of mutated genes [1]. Beyond this first generation of therapies, the currently available genome editing technologies are enabling us to obtain precise, fast and cheap modifications of human genomic sequences.

The first applicative example of the disease-modifying efficacy of these technologies was recently demonstrated in clinical trials on sickle cell disease and beta-thalassemia [2] suggesting that genome editing has a promising safety record prompting further ongoing and imminent clinical trials of in vivo genome editing [2,3,4].

As recently reviewed by Bulaklak and Gerbach [1], several experimental trials are currently addressing challenges related to genome editing safety and modelling of systems to advance in vivo genome editing to provide new clinical approaches for several human health disorders across diverse tissue types and disease conditions.

**Figure 1:**
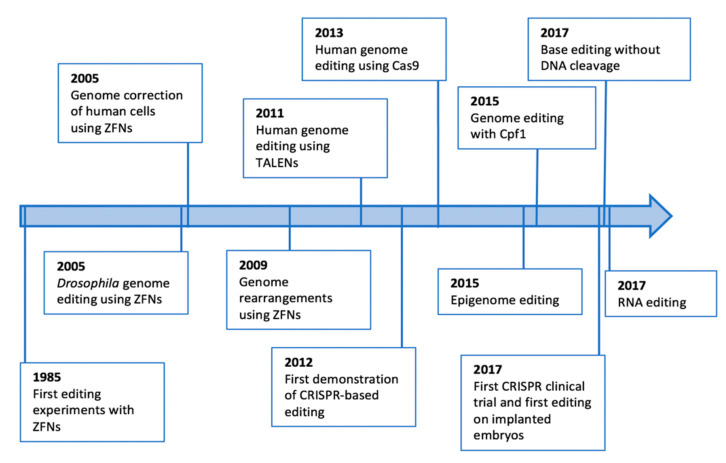
A timeline of milestones of the genome editing technologies.

In a way that is as intriguing as unprecedented in the history of life sciences [5,6], multiple next-generation editing technologies have been suggested to improve specificity, accuracy, efficiency and applicability of genome editing (Figure 1). In particular, in view of the collateral damages observed in some trials [7,8], base editing methods have developed in order to obtain precise changes in genomic sequences without inducing DNA breaks and without reliance on the activity of endogenous DNA repair pathways [6]. At the same time, RNA-targeted editing technologies have been developed, favouring transient and reversible modifications of the gene expression without inserting permanent changes into genome sequences, potentially leading to greater safety [5,9,10]. Furthermore, epigenome editing technologies have also been attempted with the advantage of increasing gene tunability without stable collateral damages. Indeed, these technologies allow the modulation of gene expression without permanently altering genomic sequences [11,12,13]. Lastly, several new, more precise and super-efficient CRISPR-Cas systems have been derived from engineered variants, various bacterial species and distinct classes of CRISPR targeting mechanisms. This enrichment in our molecular tools for genome editing allowed a true transformation of how we currently think gene therapies, broadening the number of human diseases that could be involved in clinical trials for treating them [14,15,16,17,18].

In the last years, some authors discussed genome editing technologies at a bioethical level, together with their regulatory practices [19]. However, these two very different topics have been mixed frequently, making the discussion more difficult. Indeed, the bioethics should be related to beneficence, autonomy of patients and justice that means, for instance, that if we agree that the release of these techniques will save lives worldwide, this discovery cannot be under a patent, since it would become almost or totally unattainable for many human beings [20]. We have therefore to reply primarily to bioethical questions and the regulatory practices will be successively defined to apply the genome editing techniques where it has been ethically accepted. An example of this approach is represented by genetically human embryos modified using CRISPR-Cas9 that prompted several scientists to pronounce themselves for the suspension of this type of research. If we agree with these pronouncements at the bioethical level, they can be used as arguments to regulate these novel genetic tools [21].

## 2. An Answer Expected for More Than 50 Years

In 1966, Robert L. Sinsheimer, an internationally acclaimed biologist, professor of biophysics and chancellor emeritus of the University of California, gave a talk at Caltech’s 75th Anniversary Conference about human evolution in an age of great promises about genetics [22]. In particular, more than 30 years before the publication of the genome project and more than 20 years before the first genome editing trials, he clearly observed the future of genetics: “Eventually we will surely come to the time when man will have the power to alter, specifically and consciously, his very genes. This will be a new event in the universe. No longer need nature wait for the chance mutation and the slow process of selection. Intelligence can be applied to evolution. There are those who will be concerned with the ethics of the potential modification of man, yet it seems to me this issue poses a quandary that is beyond ethics. The foundation of ethics is foresight. It is our ability to forecast the consequences of our actions that engenders our responsibility for them. But how can we possibly predict the ultimate consequence of our alteration of ourselves? Each small step will lead inexorably to another, in a cumulative, positive feedback mechanism to patterns of life and forms of knowledge and even systems of thought beyond our scope. We will have need of hope. Ours is an age of transition. After two billion years, this is the end of the beginning. It would seem clear, to some achingly clear, that the world, the society, and the man of the future will be far different from that we know. Man is becoming free, not only from the external tyrannies and caprice of toil and famine and disease, but from the very internal constraints of our animal inheritance, our physical frailties, our emotional anachronisms, our intellectual limits. We must hope for the responsibility and the wisdom and the nobility of spirit to match this ultimate freedom”.

It was a very optimistic idea of our evolution and wisdom, but Sinsheimer perfectly addressed questions that are currently present in the debate about genome editing [19].

Few months later, in 1967, the geneticist and Nobel laureate Marshall Nirenberg wrote a commentary in Science [23] about the imminent possibility of reprogramming cells to produce “synthetic” messages. In particular, he prompted scientists, politicians and society to be ready to cope with new technical improvements that will be present in the next future of genetics: “More information must be obtained before practical applications will be possible and the technical problems that must be overcome are formidable. However, when these obstacles have been removed, this knowledge will greatly influence man’s future and then man will have the power to shape his own biological destiny. The point which deserves special emphasis is thar man may be able to program his own cells with synthetic information long before he will be able to assess adequately the long-term consequences of such alterations. Long before he will be able to formulate goals and long before he can resolve the ethical and moral problems which will be raised. I state this problem well in advance of the need to resolve it because decision concerning the application of this knowledge must ultimately be made by society and only an informed society can make such decision wisely”.

The predictions of Sinsheimer and Nirenberg were really impressive since they fully met the bioethical, legal, moral and political debates that are ongoing nowadays, but at the same time they clearly highlight the impressive slowness of scientists, politicians and society in addressing these questions. Indeed, even if the development of new genome editing technologies occurred at an accelerated rate in the last decade, it is inexplicable the absence of these questions in the public agenda since the Nirenberg suggestions. At the same time scientists have not been trained to decide about the moral and political implications of their work neither the public (nor its elected representatives) has been trained to decide on scientific technologies related to genetics. As a consequence, the ongoing process that we have been driving proceeded in an ethical fashion rather than based on a rational bioethical approach.

We might be tempted to think that only Sinsheimer and Nirenberg understood the extent of twentieth-century genetics, but we would be mistaken. Indeed, less than a decade after these prophetic suggestions, the Austro-American biochemist Erwin Chargaff, still nowadays known for his Chargaff’s rule about DNA composition, wrote in his passionate autobiography entitled “Heraclitean fire: sketches from a life before nature” [24] that the humankind was near a “barrier that should have remained inviolate” and that the awesome irreversibility of the genetic engineering experiments has to be carefully evaluated: “You can stop splitting the atom; you can stop visiting the moon; you can stop using aerosols; you may even decide not to kill entire populations by the use of a few bombs. But you cannot recall a new form of life... it will survive you and your children and your children’s children. An irreversible attack on the biosphere is something so unheard of, so unthinkable to previous generations, that I could only wish that mine had not been guilty of it”. According to the Chargaff proposal, the development of DNA editing would have immense social and economic impacts demanding that we should take “a new and more critical look at the direction in which modern science and technology seem headed, at the relationship of mankind to the rest of Creation and, ultimately, they pose questions of ethics and responsibility which have been ignored in the scramble for profits and power”.

Interestingly, after the experiments performed at the J. Craig Venter Institute reporting the creation of the first bacterium with an entirely synthetic genome [25], this barrier is closer than ever. Furthermore, the nowadays situation is well beyond the concern expressed by Chargaff, since the current shift from genetic engineering to synthetic biology has considerably greater bioethical significances. Indeed, as suggested by Boldt and Müller [26], the aim of synthetic biology is not to amend an organism with a certain quantity of altered characteristics (that is, to manipulate); instead, it is to equip a completely unqualified organism with a new quality of being (that is, to create a new form of life).

In 1975, the second international conference in Asilomar (California) clearly assessed that, despite the fact that the use of technology applied to DNA was allowed in several countries, the application of genetic engineering to humanity continued to be the subject of deep debate and a growing concern was expressed more generally about the use of recombinant DNA. After about 50 years, Asilomar as a historic event seems not to have lost much of its attraction and several scientists are calling now for a new Asilomar as a symbol for the present research era [27].

As clearly shown by these examples, genetic modifications and genome editing should be well-known topics for bioethics since decades, but actually they are far from being resolved. This result certainly derives from the complexity of these topics, but it is also due the fact that we have been as addicted to effectiveness of these technologies so that the debate about these methods has been postponed for decades awaiting the discovery of breakthrough methods.

## 3. Origin, Promises and Pitfalls of the Genome Editing Technologies

The first experimental evidences of genome editing have been obtained in the 1970s and 1980s working with yeast and mouse genomes and were based on homologous recombination [28,29].

In the successive decades, it has been observed that if DNA is processed by nucleases, broken ends may be rejoined precisely, but occasionally errors are made, leading to local small insertions and deletions by non-homologous end joining [30]. At the same time, it has been also observed that DNA integrity can be restored by homology-directed repair mechanisms if a template DNA trait is furnished after DNA cleavage [30]. Despite these first intriguing results, before the advent of CRISPR-Cas9, genome editing was remarkably precise, but very inefficient.

Firstly observed as an “unusual structure” in the *E. coli* genome, the story of CRISPR began in 1987 [31], but only during the successive decades, these short regularly spaced repeats were reported in more than 40% of bacteria and 90% of Archaea and were officially named Clustered Regularly Interspaced Short Palindromic Repeats [32,33].

From 2005 to 2012 several independent research groups studied CRISPR assessing that it contains spacers of extra chromosomal origin, including spacers from phages and plasmids suggesting that CRISPR systems were a kind of bacterial adaptive immune defence mechanism that protected bacteria [34,35]. Subsequently, the discovery of the Cas gene, Cas protein, protospacer adjacent motif (PAM), CRISPR RNA and trans-activating crRNA allowed to unveil the basic function behind the CRISPR system.

In 2012, it has been observed that Cas9-crRNA complexes of the *Streptococcus thermophilus* and *Streptococcus pyogenes* could function as RNA-guided endonucleases in vitro, using small RNA molecules as guides that recognize target sequences and Cas9 to cleave DNA at specific sites [36]. Since these first findings, CRIPSR-Cas9 emerged as an efficient, versatile and robust genome editing tool for inducing specific double-strand breaks opening a new era of genome engineering for variety of cells and organisms, both in vitro and in vivo [37]. In particular, the CRISPR-Cas system can be easily programmed to specific targets differently from previous gene editing technologies, such as zinc-finger nucleases (ZFNs) and transcription activator-like effector nucleases (TALENs) requiring a re-engineering for each new DNA target [38].

At the same time, several studies demonstrated that the CRISPR/Cas9 system can induce substantial amounts of off-target mutations that can have pathogenic consequences [39]. These negative events can result in unwanted heritable genetic changes if genome editing is performed on embryos, where a low efficiency of the mutation repair has been also reported [40].

The “adult vs. embryo” comparison in editing success is particularly interesting since it shows that the same method could be differently “safe” suggesting that the effectiveness of the editing technologies cannot be the main parameter for our future choice. Indeed, safety in therapeutic alterations of the tissues of an adult might or might not be successful, but is a risk a patient or those responsible for them can reasonably assume. This is not comparable to the editing of embryos that currently cannot be reliably engineered.

In a review published in the New York Review of Books (related to four books on the prospects of using CRISPR and related gene modification technologies for the improvement of human biology), Natalie de Souza, a former Editor of the scientific journal Nature Methods, wrote that “in ten years or so, we will probably meet the minimum conditions of safety and predictability for editing out single-gene diseases” [41]. Can we really accept applying editing to human embryos when we will meet the “minimum conditions of safety”? It would probably be appropriate to cancel embryo editing from future goals to avoid confusion between applications that require different levels of safety and responsibility. This choice could also improve our chance of avoiding that the “CRISPR madness” may evolve into genetic chaos or difficulties in the acceptance of these technologies as a whole in our society. Lastly, bioethics implies that new technologies would be distributed equitably, can we really think that embryo editing (that also include the costs of the assisted reproductive technology) could fit this goal in the future?

## 4. The Role of Policy Makers and Bioethicists in the Establishment of the Limits in Genome Editing

In the last years several scientists casted doubts about the 100% effectiveness of the currently available genome editing methods, suggesting tacitly that an absolute effectiveness is *per se* a sort of synonym of acceptability of them at a bioethical level. As discussed by Gonzalez-Avila and colleagues [27], the lack of confidence in the CRISPR-Cas systems and their applications prompted the suggestion of being careful with releasing them, but none of the countries are actually thinking that laws regulating the use of these techniques should be based on a bioethical evaluation rather than on effectiveness.

The effectiveness is of course related to bioethics, but only if we already decided that we accept risks associated to genome editing so that for morality we have to properly communicate the general risks of CRISPR editing in human beings to patients. Indeed, we can agree that the premise of *primum non nocere*, which could be translated as “first, do no harm,” is useful for avoiding the improper use of these methods, but this is related to the regulatory practices rather than to bioethics. What I mean is that in the presence of 100% effectiveness we could also have ethical concerns that could be, for instance, related to unattainability of the editing methods for many human beings due to costly procedures. As suggested by Noll [42], genome editing technologies have the promise of promoting several social benefits, ranging from the reproductive freedom to the prevention and/or treatment of genetic diseases, but “these technologies could also be applied in ways that are ethically problematic, as we have learned from the history of eugenics, where advances in knowledge of genetics led to outrageous social injustices”.

The role of policy makers and bioethicists will be essential in these years to be sure of satisfying the need to benefit all the society waiting for these technologies and not only a privileged population that could pay for them. Modern science has to be focussed to reduce gaps and social inequalities instead of opening new ones [43].

The discussion about the extent of the use of CRISPR in clinical medicine frequently includes scientists asking to avoid the precautionary principle when applying such technologies for saving lives, but CRISPR-Cas is not inherently a “good” technology. As stated by Gonzalez-Avila and colleagues [27], “the new genetic modification technologies can be something applauded or something deplorable, taking into account that the tool used is not the one that determines the end, it is the user who determines the fact. After the probable approval of CRISPR-Cas as a therapeutic alternative, it is questioned how feasible it is to approve it, if it will be accessible to the public, in which cell lines it could be applied, in addition to the laws that should govern its use”.

According to some authors [44], the existing regulatory framework defined for somatic gene therapy using viral vectors should be effective at balancing scientific rigor, patient safety and innovation also for trials in genome editing since it has been honed through decades of trials. It could be true, but the regulatory framework deals about safety and it does not reply to questions related to costs, accessibility and social justice, since somatic gene therapy using viral vectors has never gained the applicative chance of current genome editing technologies.

“Should the rich be allowed to buy the best genes?” the writer Walter Isaacson asked provocatively in an essay published in 2019 [45]. In a world in which there are people who do not have access to basic medicine and general medical practices, it is hard to imagine how we will find a way to have equal access to genome editing.

I am trying to evoke scenarios like those described by Aldous Huxley in his novel “*Brave New World*”, in which the modification of embryos produced intelligence-enhanced leaders and stunted menial laborers, simply because intelligence is a multigene trait that is too complex to be engineered. However, we have to consider that public worries have been raised by editing traits not related to health, but to enhancement [44] and this distinction could be difficult to be defined. For instance, is preventing obesity a cure or enhancement? As asked by Daley and colleagues [44], what about potentially advantageous editing of genes conferring increased risks of cardiovascular disease, stroke, dementia, cancer, or susceptibility to infectious disease? We have evolved in the past few decades into an inequitable society, we have to avoid the risks that genome editing technologies could make it much worse.

Considering that several papers have been published reporting data on a large plethora of diseases that could be faced with the CRISPR-Cas technologies (including several genetic diseases, such as sickle cell anaemia, blindness, thalassemia, cystic fibrosis, hereditary tyrosinemia type I, Duchenne muscular dystrophy, mitochondrial disorders, cancer, Huntington’s disease and viral infections, like HIV, COVID, etc.) [46,47], we should be similarly active also in establishing editing limits about what will be morally and legally permissible.

Furthermore, it is also necessary to carefully evaluate the possibility that concerns related to troubling uses of genome editing methods (related, for instance, to gene enhancement or embryo editing) will compromise the public’s perception of the editing technologies also compromising their authorization for medical/health purposes.

Genome editing is a powerful technology that can reshape medical treatments and people’s lives, but we have to remind that around 65% of people have some kind of health problem as a result of congenital genetic mutations and one in five “healthy” adults carry disease-related genetic mutations [48], are we ready to edit all of them? Moreover, with the increasing success of genome editing, many more people with genetic diseases will survive to reproductive age and will ask to have also their own children without disease genes.

Recently, the Italian cellular biologists Manuela Monti and Carlo Alberto Redi [49] wrote that “philosophers, ethicists and policy makers must now communicate their opinions without generating fears and ghosts that feed the technophobic imagination of the general public, as unfortunately happened with the nuclear transplant technique that allows the generation of clones. It is time for thinkers of various backgrounds to know how to make an income from the work and years spent in the laboratory by biologists, so that medical doctors could apply optimally the results achieved and could satisfy the unanswered therapeutic demands of those who suffer today: yes, it is about understanding the difference between the technique and the product of the technique”.

I perfectly agree with them that we need an interdisciplinary discussion aimed at eliminating dogmas, misperceptions and personal prejudices, but this discussion has to be also accompanied by institutional observations and with bioethical limits established by specialized committees [50] and these arguments have to be taken seriously in the regulation of these novel genetic tools.

As scientists, we are today very lucky since we do not have to tell society what it has to decide. On the contrary, we can just kick that can into the field of “philosophers, ethicists and policy makers” asking them to decide the regulatory and bioethical frameworks, eventually complaining about their choices.

## 5. How Do We Begin to Regulate Genome Editing Technologies?

**Figure 2:**
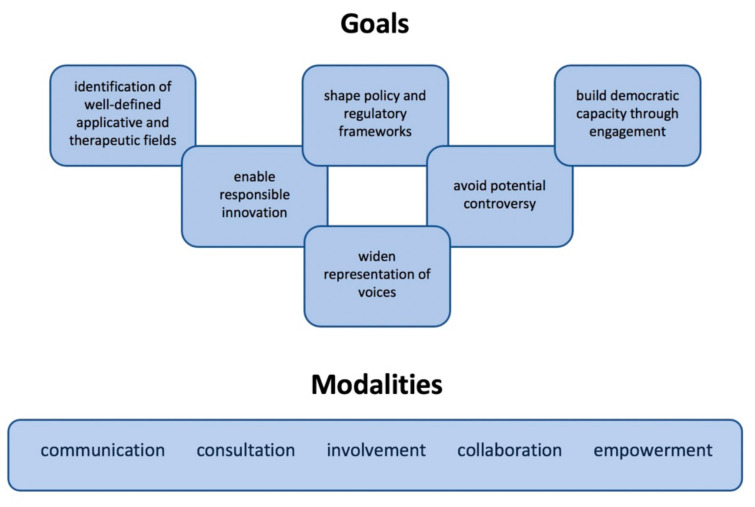
The regulatory frameworks of genome editing technologies should move from the definition of the targets of these methods (goals) and of the modalities we plan to use for building a diffuse consensus for them.

Although the safety and efficacy of embryo editing is yet to be proven, genome editing technologies can really be useful tools to eradicate various genetic diseases. At the same time, we can agree that only therapeutics purposes should be put in our agenda starting from severe monogenic diseases with serious and potentially life-threatening manifestations. Once that we agree about what we can edit has been established, it can be established how these technologies will be regulated and effectively and successfully applied (Figure 2).

As recently suggested by Townsend [50], regulation will vary between places since a normative framework is reflective of different moral codes and biotechnical approaches, but we can try to have a harmonisation of the regulatory practices.

A good starting point could be the Universal Declaration on Bioethics and Human Rights since it can “provide a universal framework of principles and procedures to guide States in the formulation of their legislation, policies or other instruments in the field of bioethics” [51]. This means that differences in the legislative, regulatory and/or policy are identified and made compatible. Harmonisation is, indeed, the result of collaboration and cooperation between jurisdictions.

This offers an opportunity to favour collaborations and synergies between international regulators, health administrators and politicians promoting an exchange in good practices that should also involve broader public consultations [50].

Considering the presence of diffuse concerns about embryos editing, we can navigate the delicate terrain of genome editing by public consultation and engagement clearly stating the purposes of the approved editing technologies and clearly defining lines that scientists should not cross assessing that “no one is playing God”.

Public engagement should involve a true consultation avoiding agenda and terms of debate poorly defined and should include focus groups, citizen juries, consensus panels, public consultations and technology assessment processes avoiding approaches that could result in a sort of “bioethical washing” [52,53]. At the same time, it is essential to avoid the idea that main reason for public hostility to technological innovation was lack of information only. As suggested by Townsend [50], the purpose of the engagement is not only to inform and educate, but also to stimulate debate and to be allowed to participate.

Lastly, it is difficult to examine society’s acceptance or rejection of key biotech technologies without considering the role played by the world’s major religions and their belief structures, since each opinion is formed within a particular philosophical, cultural, religious and social context. These differences are not a limit, by they should be welcomed since they can improve the discussion [50,54].

## 6. Conclusions

The work of scientists in the laboratories provided us with the ability to permanently alter our DNA, we now deserve the opportunity to work together to reach some consensus in order to handle genome editing techniques (not to prohibit them) with care, because these techniques can really eradicate genetic diseases.

At the same time, considering that we will probably not have a common regulatory framework for genome editing fitting all countries, it is essential that genome editing technologies must be regulated through a combination of national and supranational legislative regulation or ‘hard’ law, in combination with ‘soft’ ethics, firmly anchored in and underpinned by human rights values. As we already diffusely observed, ethical values are dynamic and ethical thinking evolves in some countries faster than in other ones. Similarly to what occurred with Agenda 2030 for sustainability, we should ask for a general agreement about the applicative fields of the editing techniques (e.g., prohibition of modification of embryos) with specific national laws precisely regulating them in each country. This is essential to avoid that inequality could results from economic incomes of people who can move to less stringent countries where editing is applied more diffusely favouring a sort of genetic tourism.

We therefore have to ask for worldwide regulatory frameworks endorsing values and universally accepted human rights: the right to life, the rights of the future child, the right to health care, the right to dignity, the right to equality and non-discrimination, and the freedom of scientific progress. As recently discussed by Townsend [50], these positions have already been discussed in several declaration, such as the Universal Declaration on the Human Genome and Human Rights, the International Declaration on Human Genetic Data and the Universal Declaration on Bioethics and Human Rights, so that we have to start to define normative framework within which scientists may (or may not) lawfully and ethically operate: “what is clear, however, is that we cannot allow rogue actors to make these irreversible decisions for us all”.
